# Reward Salience and Choice in a Controlling Context: A Lab Experiment

**DOI:** 10.3389/fpsyg.2022.862152

**Published:** 2022-04-25

**Authors:** Rosa Hendijani, Piers Steel

**Affiliations:** ^1^Faculty of Management, University of Tehran, Tehran, Iran; ^2^Haskayne School of Business, University of Calgary, Calgary, AB, Canada

**Keywords:** performance-contingent reward, reward salience, choice, performance, motivation

## Abstract

One of the challenges in the motivation literature is examining the simultaneous effect of different motivational mechanisms on overall motivation and performance. The motivational congruence theory addresses this by stipulating that different motivational mechanisms can reinforce each other if they have similar effects on the perceived locus of causality. Reward salience and choice are two motivational mechanisms which their joint effects have been long debated. Built upon the motivational congruence effect, a recent empirical study affirms that a salient reward in a condition characterized by lack of choice and a non-salient reward in a condition characterized by provision of choice both increase overall motivation and performance. In this study, we examine the effect of reward salience and choice on overall motivation and performance in a controlling context, an effect which has not been studied before. A 2 (choice: present, absent) × 3 (reward: salient, non-salient, none) factorial design was conducted to examine research hypotheses. The results show that under controlling conditions, salient reward improves overall motivation and performance compared to non-salient and no-reward conditions.

## Introduction

Different motivational mechanisms operate not only alone but in combination with each other to influence overall motivation and direct behavior (Schwartz and Wrzesniewski, 2016; Woolley and Fishbach, 2018). One of the main challenges of the motivation literature is to examine the dynamics of such interactions and their effect on overall motivation (i.e., the sum of different types of motivations) and performance (Bonner and Sprinkle, 2002; Cerasoli et al., 2014; Goswami and Urminsky, 2017; Wowak et al., 2017; Woolley and Fishbach, 2018; Hendijani and Steel, 2020). Among these, the combined effect of external reward and choice has been particularly controversial (Patall et al., 2008; Hendijani, 2021). This is mainly due to the contrasting views of two major theoretical streams in the motivation literature, including behaviorism and cognitive evaluation theory (Hendijani and Steel, 2020). The purpose of current study is to examine the effect of reward salience and choice on overall motivation and performance in a controlling context. This effect has not been previously studied in the literature.

From the perspective of behaviorism (Skinner, 1953; Vroom, 1964; Vroom et al., 2005) and standard economic theories (Lazear, 1999, 2000), motivational mechanisms are generally independent and reinforce each other’s effect (Steel and König, 2006). Here, the two mechanisms of external reward and choice can be additive and positively influence motivation and performance. In contrast, theories of cognitive evaluation (Deci, 1971; Deci et al., 1999), self-determination (Ryan and Deci, 2000, 2019), overjustification effect (Levy et al., 2017), and motivational crowding-out (Frey and Jegen, 2001) argue that certain types of external rewards, especially performance-contingent ones can be perceived as controlling. Therefore, they negatively interact with choice and undermine overall motivation and performance (De Charms, 1968; Patall et al., 2008; Ryan and Deci, 2019). Several mediating factors, including task type (Gagné and Deci, 2005), reward salience (Ross, 1975; Beus and Whitman, 2017), expectancy (Greene and Lepper, 1974), and level of intrinsic interest toward the task (Kruglanski et al., 1975) have influenced the predictions and empirical results related to these two theoretical streams (Lacetera et al., 2012; Madden et al., 2013). Among these factors, the notion of reward salience is captured and highlighted by the attributional theories (Bem, 1967; Kelley, 1973). According to these theories, the salience of reward rather than its mere presence can result in an extrinsic perceived locus of causality that can negatively interact with choice and diminish overall motivation and performance (Nisbett and Valins, 1971; Kruglanski et al., 1972; Ross, 1975).

In line with the attributional framework, motivational congruence theory addresses the joint effect of different motivational mechanisms from the perspective of their influence on the perceived locus of causality (Hendijani and Steel, 2020; Hendijani, 2021). Accordingly, different motivational mechanisms, regardless of being intrinsic or extrinsic, can reinforce each other’s effect if they can create consistent effects on the perceived locus of causality. Such consistency results in an overall intrinsic/extrinsic motivation and improves performance. On the other hand, they can cancel or mitigate each other’s effect if they affect the individual’s perceived locus of causality in opposing directions. Such contrasting effects can result in a state of confusion in the perceived locus of causality, which in turn attenuates overall motivation and performance.

Consistent with motivational congruence effect, a recent experimental study examined the effect of reward salience and choice on overall motivation and performance. The results showed positive interactions between non-salient/salient reward and choice/no-choice on overall motivation and performance (Hendijani and Steel, 2020). These results highlight the importance of reward salience. Despite its central role (Hewett and Conway, 2016; Berridge, 2018), salience has not been sufficiently considered in previous studies examining the simultaneous effect of external reward and choice on motivation and performance (e.g., Cohen, 1974; Hallschmid, 1977; Bartleme, 1983; Margolis and Mynatt, 1986; Marinak, 2004).

In this study, these effects are studied in a controlling context. According to the literature, a controlling context can be characterized by factors such as effortful and attention-demanding tasks (Mulligan and Hartman, 1996; Mulligan, 1997), time pressure, and deadlines (Amabile et al., 1976; Ryan and Deci, 2000; Muraven et al., 2008). The results indicate that salient reward improves overall motivation and performance compared to non-salient and no-reward conditions, while choice has no effect. These results are consistent with the motivational congruence theory and highlight the importance of a match between type of motivational mechanism and the environment within which it is administered.

## Literature Review

**FIGURE 1:**
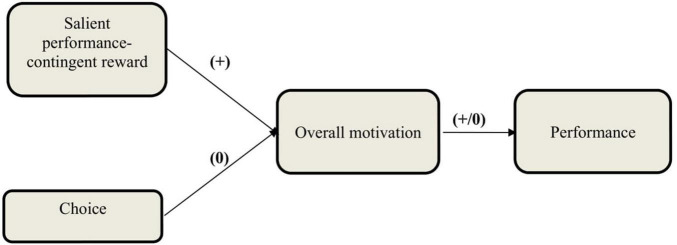
Effect of reward salience and choice on overall motivation and performance in a controlling context.

Reward (Roth et al., 2007; Garbers and Konradt, 2014) and choice (Cordova and Lepper, 1996; Williams, 1998; Gagné and Bhave, 2011; Patall, 2013) are two motivational mechanisms that have been widely used in a variety of settings, including organizational and educational settings. A few studies in the past examined their simultaneous effect on motivation and performance (e.g., Cohen, 1974; Hallschmid, 1977; Bartleme, 1983; Margolis and Mynatt, 1986; Marinak, 2004). In addition, one study examined the role of reward salience as a factor that influences the simultaneous effect of these two motivational mechanisms on motivation and performance (Hendijani and Steel, 2020). In the current study for the first time, the effect of reward salience and choice on overall motivation and performance is studied in a controlling context. In line with motivational congruence theory, it is hypothesized that the effect of choice is neutralized by the controlling nature of the environment while salient reward improves overall motivation and performance. Figure 1 depicts the schematic diagram of the research hypotheses for this study.

### Interaction Between Different Motivational Mechanisms

One of the main challenges in the motivation literature is to examine and predict the simultaneous effect of different motivational mechanisms on overall motivation and performance. Overall motivation is defined as the sum of different types of motivations, including both intrinsic and extrinsic motivations (Hendijani et al., 2016). The issue became more prominent with the cognitive evaluation theory’s dichotomization of intrinsic and extrinsic motivations (Deci, 1971). The three streams of reinforcing, undermining, and contingency theories of motivation have made contradictory predictions regarding the combined effect of different motivational mechanisms (Hendijani and Steel, 2020; Hendijani, 2021). Among these interactions, the joint effect of external rewards as one major type of extrinsic motivational mechanisms and other intrinsic motivational mechanisms (e.g., the provision of choice) has been highly debated over the past few decades (e.g., Calder and Staw, 1975; Kvaløy et al., 2015; Hendijani et al., 2016). In the next sections, we review the literature related to the external reward and choice as the main study variables and develop the research hypotheses.

### External Rewards

The main theoretical streams in the motivation literature have made different predictions regarding the effect of rewards on overall motivation and performance. Reinforcing theories of motivation, such as expectancy-value theories (Vroom, 1964; Vroom et al., 2005) and theory of learned industriousness (Eisenberger, 1992; Eisenberger and Cameron, 1996) predict that motivational mechanisms are generally additive and can independently improve overall motivation and performance (Eisenberger and Cameron, 1996; Porter and Lawler, 1968). According to these theories, the provision of external rewards both separate or in combination with other motivational mechanisms can improve motivation and performance. This can happen through several mechanisms, such as increasing task value (Furnham and Argyle, 1998), estimates of success (Locke and Latham, 1990, 2019; Wright and Kacmar, 1995; Bonner and Sprinkle, 2002), and perceived competence (Eisenberger and Cameron, 1996). Several empirical studies (Eisenberger and Cameron, 1996; Eisenberger and Aselage, 2009; Hennig-Schmidt et al., 2011; Lacetera et al., 2012; Grandey et al., 2013; Stazyk, 2013; Hendijani et al., 2016) and meta-analytical reviews (Byron and Khazanchi, 2012; Cerasoli et al., 2014; Garbers and Konradt, 2014) support these predictions by finding positive effects of external rewards on motivation and performance.

In contrast with reinforcing theories’ predictions, undermining theories, including cognitive evaluation theory (Deci and Ryan, 1985; Deci et al., 1999), self-determination theory (Ryan and Deci, 2019), and motivational crowding-out (Frey and Jegen, 2001; Weibel et al., 2014) argue that extrinsic rewards, especially performance-contingent ones can undermine intrinsic motivation due to their controlling characteristic. According to these theories, intrinsic motivation is built upon two underlying needs of autonomy and self-competence. Since rewards, especially performance-contingent ones, can control and direct behavior, they can inhibit autonomy and undermine intrinsic motivation. According to these theories’ prediction, the provision of reward along with other intrinsic motivational mechanisms (e.g., the provision of choice) can mitigate their positive effects and diminish motivation (Patall et al., 2008). While empirical studies (Lepper et al., 1973) and meta-analytical reviews (Deci et al., 1999) have supported these theories’ predictions, there are several moderating factors related to the design and implementation of these studies which could influence the results and make them difficult to interpret (Cameron et al., 2001; Cameron and Pierce, 2002; Madden et al., 2013; Woolley and Fishbach, 2018). The use of games instead of real-world tasks (e.g., Enzle and Ross, 1978; Hamner and Foster, 1975) and reward salience (e.g., Deci, 1971; Lepper et al., 1973; Calder and Staw, 1975; Anderson et al., 1976) are two commonly addressed design issues related to these studies that confound the results and limit their interpretations (Hendijani et al., 2016; Woolley and Fishbach, 2018).

As the third category, contingency theories highlight the role of self-perceptions of the locus of causality regarding the effect of different motivational mechanisms on overall motivation and performance. Attributional theories are the main theories in this category. Instead of the actual cause of a behavior, these theories focus on the attribution made by the individual regarding the cause of a behavior (Ross, 1975; Ferrin and Dirks, 2003) and highlight the role of perceived motivational orientation (i.e., locus of causality), which refers to the individual’s interpretation of the cause of own motivation and behavior. If individuals attribute their motivation and behavior to an internal (external) factor, they feel they are intrinsically (extrinsically) motivated. These theories focus on the notion of reward salience and argue that a salient reward can increase the likelihood of an extrinsic perceived locus of causality and conduce to extrinsic type of motivation, while a non-salient reward can contribute to an intrinsic perceived locus of causality and result in an intrinsic type of motivation. Reward salience refers to the conditions under which reward becomes a focal consideration in the mind of the individual. Under such conditions, the individual tends to think about the reward, become motivated to gain more rewards or make sure the flow of the reward is unobstructed (Beus and Whitman, 2017). Thus, according to these theories, the salience rather than the presence of external rewards can contribute to the negative interaction effects observed in the undermining literature (Ross, 1975).

In line with contingency theories, the motivational congruence effect (Hendijani and Steel, 2020) was developed, which predicts that the effect of different motivational mechanisms regardless of being either internal or external depends on their congruence or incongruence with each other and the environment in which they are administered (Hendijani, 2021). Consistent with this theory, empirical study indicated that the provision of a salient reward in a context characterized by the lack of choice created an extrinsic perceived locus of causality and improved overall extrinsic motivation and performance. In contrast, the provision of a non-salient reward in a choice condition resulted in an intrinsic perceived locus of causality, which improved overall intrinsic motivation and performance. However, the provision of a salient/non-salient reward in a no-choice/choice condition produced tension in the perceived locus of causality and decreased overall motivation and performance (Hendijani and Steel, 2020). Therefore, the notion of salience can play an important role in the combined effect of external rewards and other motivational mechanisms and should be considered when examining the joint effect of external rewards and other types of motivational mechanisms on overall motivation and performance.

### Choice

Choice refers to “the act or opportunity of choosing” (Merriam-Webster, 2022). Many studies suggest that people may feel more motivated and perform better when they can make choices (Iyengar and Lepper, 1999; Patall et al., 2008). It can create a feeling of “personal causation” and improve persistence in a task and satisfaction with its consequences (Lewin, 1952; De Charms, 1968). The effect of choice on motivation and performance has been widely discussed in the literature (Enzle and Anderson, 1993; Nie et al., 2015). According to self-determination and cognitive evaluation theories, autonomy is of the main pillars of intrinsic motivation (Ryan and Deci, 2000). Choice provision is one of the external mechanisms that can support the feelings of autonomy (Patall, 2012). It has been widely used to motivate individuals in a variety of contexts, including educational or work contexts to create an autonomy-supportive condition and enhance intrinsic motivation (Patall, 2012, 2013). However, empirical studies on choice have found mixed results regarding its effect on motivation (Patall et al., 2008). While some studies found positive effects (e.g., Cordova and Lepper, 1996; Weinstein and Ryan, 2010), others found either a few positive effects (Flowerday and Schraw, 2003), negative effects (Flowerday et al., 2004), or no effects (Overskeid and Svartdal, 1996; Reeve et al., 2003) on motivation and other related performance outcomes.

Several factors might contribute to the differences in the effect of choice. First, studies with negative or no effects have mainly examined the effect of choice making in one task on the level of effort and persistence in a subsequent task, which is usually different from the choice-making activity. Such designs can result in a condition of ego-depletion or mental fatigue due to the choice making activity that lead to a lack of motivation and performance in the subsequent task (Patall et al., 2008). The second factor is related to the use of a controlled vs. an autonomy-supportive condition in the manipulation of choice. In controlled conditions, individuals are subtly pushed to choose a target activity over other options. In contrast, in autonomy-supportive conditions, individuals are able to make unrestricted choices without being pressured to select any specific options. Controlled conditions of choice provision can lead to an environment of pressure that may neutralize or reverse the positive effect of choice on motivation and performance (Moller et al., 2006).

### Reward Salience and Choice in a Controlling Context

In this section, we develop hypotheses regarding the effect of reward salience and choice under controlling conditions. Controlling condition is defined as “the purposive influence on the regulation of an individual’s behavior through hierarchical authority which leads to the attainment of institutional goals” (Fayol and Urwick, 1963; Weibel et al., 2014, p. 79). The main purpose of control is to encourage an extrinsic motivation toward specific goals or tasks (Weibel et al., 2014). As several scholars argue, control is naturally in contrast with autonomy (Argyris, 1957; Walton, 1985). Therefore, it can undermine autonomous motivation (Weibel, 2010). Under controlling conditions, the effect of choice can be attenuated or canceled out.

Previous empirical studies have shown that controlling conditions, such as time and performance pressure (Amabile et al., 1976; Deci et al., 1994; Ryan and Deci, 2000; Muraven et al., 2008), surveillance (Enzle and Anderson, 1993), and provision of controlled choice (Moller et al., 2006) can indeed undermine perceptions of autonomy and neutralize (Overskeid and Svartdal, 1996; Reeve et al., 2003) or reverse the effect of choice (Flowerday et al., 2004; Ryan and Deci, 2019). Therefore, we develop the following hypotheses:

H1a: Under controlling conditions, choice does not have an effect on overall motivation.
H1b: Under controlling conditions, choice does not have an effect on performance.

In contrast, salient reward would match a low choice environment due to its controlling nature. Thus, administration of salient reward in a controlling environment can result in an extrinsic perceived locus of causality (Deci and Ryan, 1985, 1987), leading to an extrinsic or controlled type of motivation and performance improvement. Previous empirical studies have provided support for the positive effect of salient rewards on performance in these types of conditions (Jensen et al., 2007; Hickey et al., 2010; Madan and Spetch, 2012).

H2a: Under controlling conditions, salient reward improves overall motivation compared to non-salient reward condition.

H3a: Under controlling conditions, salient reward improves overall motivation compared to no reward condition.

Considering that performance improvement is mediated by the improvement in overall motivation, we have the following hypotheses regarding the role of overall motivation:

H2b: Under controlling conditions, salient reward improves performance compared to non-salient reward condition through the mediating effect of overall motivation.

H3b: Under controlling conditions, salient reward improves performance compared to no reward condition through the mediating effect of overall motivation.

## Materials and Methods

In order to test the research hypotheses, a lab experiment was designed and conducted. Prior to conducting the study, it was reviewed and approved by the Conjoint Faculties Research Ethics Board (CFREB) at the related university.

### Experimental Task and Participants

We used a free recall memory test, as per Mulligan and Hartman (1996). Participants were first presented with a set of words and then asked to memorize and write down as many of them as they remembered (Brandt, 1991). Participants were 195 undergraduate business school students at a medium-sized Canadian university (94 males and 101 females). They completed the experiment for a 2% bonus mark in one of their general courses. The participants were 20.35 (*SD* = 2.32) years old on average with an age range between 18 and 40 years old. They were randomly assigned to one of the experimental conditions. An *a priori* power analysis showed that a sample size of approximately 195 could give us a power of 84% to detect a minimum effect size of 0.055 for reward (three levels: salient, non-salient, no reward) and a minimum effect size of 0.045 for choice (two levels: choice and no-choice) variables (Faul et al., 2009). These effect sizes are consistent with similar studies on reward (e.g., Hendijani and Steel, 2020) and choice (e.g., Patall et al., 2008) and their effect on motivation and performance.

### Experimental Procedure

This experiment was a 2 (choice variable: choice and no-choice) × 3 (reward: non-salient, salient, and no reward) between-subject factorial design. The use of a between-subject design has several advantages compared to a within-subject design. First, it does not allow the participants to guess the purpose of the study by being faced with different conditions. Second, it prevented from carry-over effects from other conditions because the participant experiences only one condition. Carryover effects refer to effects such as fatigue or practice that can improve or decrease performance in all participants, disregarding their experimental condition. Some of the more problematic types are differential carryover effects, where the carryover depends on the condition that is first experienced by the participant. Finally, in some studies, the use of within-subjects designs are not feasible. For example, in memory studies, participants may learn a strategy in the initial condition which can influence their performance in the subsequent condition. Therefore, the researcher cannot consider them as control cases in subsequent conditions (Maxwell et al., 2017).

At first, participants answered a series of questions to measure and control for their interest, competence, and confidence in their performance in a general memory test. Then, participants were randomly assigned to one of the six treatment conditions in which the choice and reward salience was manipulated based on their experimental condition. After the manipulation was complete, they completed an online memory test. After the memory test, all participants completed a set of final set of questions, which included items related to choice and reward salience manipulation check, self-reported overall motivation, and demographics. Then, participants received feedback regarding their performance in the memory test at the end of the session, after all other assessments. No performance feedback was provided during the experimental session. Participant in the pay conditions were then guided to a separate room to receive their payment. Participants in both non-salient and salient pay treatments received a performance-contingent payment of 25/ c for each correctly memorized word. The experiment was implemented on MediaLab, a software that is used for designing and conducting laboratory experiments.^1^

**FIGURE 2:**
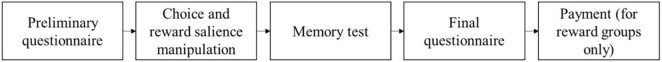
Experimental procedure.

The flow chart of the experimental procedure is presented in Figure 2.

The materials used for the memory test were based on established methods used in previous related studies (e.g., Graf et al., 1985; Rappold and Hashtroudi, 1991). The general method includes presenting a set of words to participants in a specific time frame and asking them to memorize and write as many words as they could remember in a short time span after the words were presented. This procedure is referred to as the free recall memory test (Madan and Spetch, 2012). In total, participants completed 7 trials of a 12-item free-recall memory test. In each section, they saw a set of 12 words that were randomly selected from different word categories. The selected words were among the 16 categories developed by Battig and Montague (1969) norms, including sports, fruits, vegetables, birds, pieces of furniture, colors, animals, pieces of clothing, trees, musical instruments, parts of the human body, dances, insects, food flavoring substances, fish types, and parts of a building. They were randomly selected from different categories, but were presented to all participants in the same order. Following previous studies, we selected the items that did not rank in the 10 most frequent instances, but were remembered by at least 10 out of the 400 participants in the Battig and Montague norms (Mulligan and Hartman, 1996). The words were presented at a rate of 3 s per item (Rappold and Hashtroudi, 1991). The average rank of the items selected from each category was 21.6 (range: 11–40).

In order to make the task environment controlling, we incorporated several elements in the experimental design. First, we chose a type of task (i.e., an explicit free recall memory test) that needed high attention during the first phase (i.e., word presentation phase) and demanded a controlled and conscious information processing during the second phase (i.e., recall period) (Mulligan and Hartman, 1996; Mulligan, 1997). Compared to implicit memory tests, which require an automatic recollection of knowledge, these tests are more effortful and attention demanding and require controlled information processing (Ackerman, 1987; Mulligan, 1997). Second, in each test trial, participants first saw a set of 12 words that appeared on the screen only for 3 s. They were not allowed to click or change the rate or speed of word presentation at this stage. This decreased the feelings of autonomy because they could not proceed through the test at their own pace. Forcing participants to do a task quickly compared to letting them work at their own pace has been used in previous studies as part of the manipulation for increasing the level of environmental control (Amabile et al., 1976).

Third, after finishing each set, participants were given 3 min to type all the words they memorized from the related section. During the response time, a sentence appeared and remained on top of the screen to make the time salient. The sentence was: “You have a **maximum of 3 min**. Please type as many words as you remember. Press Continue after typing each word.” Based on previous studies, time pressure and deadlines can diminish the feelings of autonomy and make the task to be perceived as controlling (Amabile et al., 1976; Ryan and Deci, 2000; Muraven et al., 2008). By applying these features, we designed this task to reduce autonomy compared to the cognitive task used in Hendijani and Steel (2020). We later confirm this with our manipulation checks of the controlling conditions.

It is important to differentiate the no-choice condition and the controlling context. Provision of choice is an external motivational mechanism that is used to enhance the feelings of autonomy and improve motivation and performance (Patall, 2013). Thus, a choice/no-choice condition is a condition in which the person has/has not received such motivational mechanism of having choice. A controlling context is the broad manifestations of situations (e.g., structured task and time pressure) where people feel they lack autonomy in their task or the environment with which they interact (Weibel et al., 2014).

### Study Variables

Overall motivation was measured by both a behavioral measure and self-reported measure. The behavioral measure was calculated by the amount of time spent during the free recall period where participants typed their memorized words. Since there were 3 min given for recall in each trial, the maximum amount of time was 21 min in the 7 trials. The self-reported measure of overall motivation had 11 items which were measured on a 7-point Likert scale. They were selected from the Intrinsic Motivation Inventory (IMI) (Ryan et al., 1991) and have been widely used in previous studies of motivation (e.g., Plant and Ryan, 1985). The items measure several aspects of motivation, including interest/enjoyment, value, and perceived effort. Following principal component analyses, the two measures were later combined in order to capture both the quantitative and qualitative aspects of motivation (Hendijani et al., 2016). The results of the principal component analyses are reported in the overall motivation measurement section. Performance was measured based on the total number of correctly memorized words.

The study also included several control variables including confidence, competence, interest, and pure guessing. The first three control variables were measured prior to the test, while the last one was measured after the memory test. Participants were asked to respond to the related questions on a 7-point Likert scale ranging from 1 (not at all) to 7 (very much). These control variables are assessed and included in the study in order to avoid any probable confounding effect of these factors on motivation and performance (Campbell et al., 1993). Items related to these variables were adapted from previous studies (e.g., Hendijani et al., 2016; Hendijani and Steel, 2020). Interest was measured with two items related to participants’ general interest in taking memory tests (“1. Please indicate how interested you are in taking memory tests” and “2. Please indicate how interested you are in memory tests in general.”). Competence was measured with two items (“1. Please indicate how you evaluate your memory.” and “2. Please indicate how good your memory is.”). Confidence was measured with one single item (“In the next sections, you will take a set of memory tests. Please indicate how confident you are that you will perform well in these tests.”). Pure guessing was measured by asking participants to indicate the percentage of the answers given based on pure guessing.

### Choice Manipulation

The choice manipulation was done prior to the experimental task. Participants were randomly assigned to either the choice or no-choice condition. We followed the manipulation used in the prior study done by Hendijani and Steel (2020). In the choice condition, participants were given full choice to select their test, while in the no-choice condition, the experimenter selected the test for them. Participants in both choice and no-choice conditions took very similar tests with a few minor differences. Supplementary Appendix I provides the manipulation for choice/no-choice.

This manipulation had several advantages. First, participants in the choice and no-choice conditions completed approximately *similar tasks*. Task similarity removed the confounding effect of abilities and skills that influenced previous choice manipulation methods. Second, this manipulation prevented from discarding data or using pressured choice, which happened in studies that used a matched design. This allowed for a state of autonomous choice because participants were not forced to choose one task over another. Therefore, choice was true and meaningful. Finally, the two options were equally interesting which avoided confounding choice with interest.

### Reward Salience Manipulation

In order to manipulate reward contingencies, participants were randomly assigned to one of the three conditions, including salient reward, non-salient reward, and no reward. Reward contingencies were indicated both in the written consent form and at the beginning of the memory test. As mentioned, participants in both non-salient and salient reward conditions received the same amount of 25/ c per correct answer in the memory test. To manipulate reward salience, participants in the salient conditions saw a picture of a set of several $100 notes with the following explanation on their computer screen at the beginning of the memory test: “You will earn a lot of money if you perform well in the memory test.”

One advantage of this type of salience manipulation is that the reward value is exactly the same (25/ c per correct answer) in the salient and non-salient reward conditions. The only difference is how the reward is presented. This manipulation is used in line with previous studies suggestion that reward salience can be operationalized by making the reward conspicuous and perceptually (e.g., visually) prominent (Taylor and Fiske, 1978; Hewett and Conway, 2016). Similar methods have been used in previous experimental studies (e.g., Lepper et al., 1973; Ross, 1975; Anderson et al., 1976). In addition, due to random assignment, the participants in the two groups were not systematically different in how they perceived the value of money (Maxwell et al., 2017).

As participants in the reward salient conditions continued the test, they saw an amount of money called *potential earning* at the button of the screen on the left corner. It continuously reminded the participants of the money that they could earn if they could correctly memorize all the words to that point. For example, for the third word in the memory test, the person saw a potential earning of 75/ c (i.e., 3*25/ c) on the screen, indicating that the person could potentially earn 75/ c if he/she could correctly memorize the words to that point.

Since potential earning was presented in a box at the bottom of the page in the salient conditions, it was possible that it could distract participants from the main experimental task. In order to create the same level of distraction in non-salient reward and no-reward conditions, participants in these groups were also shown a number called *test progress* instead of potential earning at the bottom left corner of their screen. Test progress and potential earning provided approximately identical information and levels of distraction (Hendijani and Steel, 2020).

## Results

### Manipulation Checks

In order to ensure that the manipulations for reward salience and choice worked well, a series of related questions were asked after the memory test (Hendijani and Steel, 2020). For choice manipulation, participants were asked two questions: (1) I felt like it was NOT my own choice to take this test, and (2) I was NOT given a choice to select the type of memory test. Using the average score on these items, we created a variable called ChoiceGrade. The regression of choice on ChoiceGrade showed that participants in the choice conditions perceived significantly higher level of choice in comparison with the participants in the no-choice conditions (*B* = 2.70, *p* < 0.001), so manipulation for choice worked. In addition, the percentage of selection of the memory test type A vs. B in the choice condition showed no significant difference. It was approximately 48%, which indicates that people chose the two options approximately with the same rate and were not biased toward selecting one option over the other.

In order to check whether the manipulation for reward salience had worked, participants in the reward conditions answered two salience-related questions: (1) Money was very much highlighted in this experiment and (2) Monetary reward was mentioned repeatedly during the experiment. Averaging the two responses, we created a variable called Salience. The results of the regression on the relationship between Salient reward/Non-Salient reward treatment on Salience show that our manipulation for reward salience was effective; participants in the salient reward conditions perceived significantly higher emphasis on reward in comparison with participants in non-salient reward conditions (*B* = 3.15, *p* < 0.001).

To check whether task context in this experiment was more controlling compared with the math test conducted in a previous study conducted by Hendijani and Steel (2020), a pre-test was designed and conducted with the two tasks. About 50 participants were randomly assigned to two conditions in which they completed either the memory or the math test. After completing the tasks, participants answered two questions: (1) I did this activity with my own autonomy, and (2) I did this activity because I had no autonomy (R). The variable Autonomy was calculated by finding the average score for the reversed and direct items. The result of the regression of task type (math test = 1, memory test = 0) on the variable of Autonomy showed a significant positive effect (*B* = 6.12, *p* < 0.001). Thus, consistent with our design features, task context was significantly more controlling compared to the previously used math test context.

### Overall Motivation Measurement

Overall motivation is a multi-faceted construct. It has often been assessed with two main measures of time spent on the task (i.e., the behavioral measure) and self-report of interest and enjoyment (i.e., self-reported measure). In line with previous studies (Deci et al., 1999; Hendijani et al., 2016; Hendijani and Steel, 2020), we used both measures to have a well-rounded assessment of overall motivation. From a theoretical perspective these measures capture different facets of the construct of overall motivation. Therefore, we combined them into a composite measure of overall motivation.

Composite measures are generally used to increase reliability and decrease measurement error through multivariate measurement (Hair et al., 2014). In this approach, the researcher does not use only a single variable to represent a multi-dimensional concept. Instead, the researcher measures the concept with several indicators, all representing differing facets of the construct and then combines them to create an overall construct (Hair et al., 2014).

To test whether we could combine the scales and create a composite measure, we conducted a series of principal component analyses. First, a principal components analysis with Varimax rotation was conducted on the self-reported measure. This resulted in a 3-item factor for interest/enjoyment, a 2-item factor for perceived effort, and a 2-item factor for value. Information about the questions and the factor loadings are provided in Supplementary Appendix II. To obtain one single measure for self-reported overall motivation, we converted the items into three factors of interest/enjoyment, perceived effort, and value by calculating the average value of the related items. A principal component analysis on these three factors resulted in one extracted component. The Eigenvalue for the first component was 1.82 (>1) and for the second one was 0.68 (<1). In addition, the first component accounted for 61.50% of the variance, whereas the second component accounted for only 22.00% of the variance. In addition, the components matrix results showed loadings of 0.86 for interest/enjoyment, 0.86 for perceived effort, and 0.80 for value. These results indicate that there is a strong correlation between the three dimensions of interest/enjoyment, perceived effort, and value and the underlying construct of self-reported overall motivation. These results support combining these variables to create a single measure of self-reported overall motivation.

To examine whether we could combine the self-reported and behavioral measures, we converted the two measures into a common metric. Each measure was changed into a percentage value by dividing by its maximum. A principal component analysis on these two variables resulted in one extracted component. The Eigenvalue for the first component was 1.21 (> 1) and for the second one was 0.79 (< 1). In addition, the first component accounted for 60.29% of the variance, whereas the second component accounted for only 39.70% of the variance. The results of the components matrix also showed that the loadings for both measures were 78%. This indicated that there was a strong correlation between the two measures and the underlying construct. Considering these results, we combined these two measures to create one composite measure of overall motivation.

### Descriptive Statistics and Correlations

There was not a significant difference in the number of females and males in different treatment conditions. Participants were equally divided between the six conditions with each condition having approximately 32 participants. The mean values for overall motivation and performance are highest in the no-choice/salient and choice/salient conditions, respectively. Table 1 presents the descriptive statistics for the control and outcome variables. Control variables include confidence, competence, interest, and pure guessing (PureGuess). No significant difference is found between the participants in the six experimental conditions, regarding the control variables. In addition, no significant difference is detected between females and males on the control variables.

**TABLE 1:** Descriptive statistics for control and outcome variables.

Condition	Min	Max	Mean	*SD*
Performance	0	66	40.49	10.91
Overall (behavioral)	1.69	13.25	6.12	2.54
Overall (self-report)	2	6.78	4.31	0.83
Overall (composite%)	19.00	57.00	36.12	6.69
Confidence	1	7	4.45	1.18
Competence	1	7	4.76	1.12
Interest	1	7	4.69	1.43
PureGuess (%)	0	95	23.12	20.61
Valid N	195	195	195	195

Table 2 presents the correlations between the outcome variables. As the results indicate, there is a high correlation between the three measures of overall motivation and between overall motivation and performance.

**TABLE 2:** Pearson’s correlations between outcome variables.

	1	2	3	4
1. Performance	–			
2. Overall (behavioral)	0.41*** (0.00)	–		
3. Overall (self-report)	0.26*** (0.00)	0.23*** (0.00)	–	
4. Overall (composite)	0.43*** (0.00)	0.52*** (0.000)	0.95*** (0.000)	–

*** p< 0.001

### Mediation Models

We conducted the mediation analyses both with and without control variables. The results were robust and conclusions were consistent. The use of a mediation test with bootstrapping technique has several advantages. First, it allows us to test both the direct and indirect effects of reward salience and choice on overall motivation and performance in one single model (Hayes, 2013). Second, the method is based on Bootstrap confidence interval which does not require the normality assumption. Third, it has high power and is able to detect statistically significant results even in small samples (Shrout and Bolger, 2002; Fritz and MacKinnon, 2007).

It is important to note that while the study had a 2 × 3 experimental design, no interactions were predicted according to the study hypotheses. Therefore, simple mediation tests without interactions are reported in the paper. However, the interactions tests were separately conducted and the results were not significant which are consistent with the study hypotheses.

For simplicity, we report the results with control variables in the main text. The results of the mediation tests without control variables are provided in Supplementary Appendix III. To test hypotheses H1a and H1b, we ran a mediation model with a bias-corrected bootstrap with 95% confidence intervals (5,000 bootstrap samples), using the PROCESS macro (Preacher et al., 2007). In this model, choice is set as the independent variable and other variables are added as the covariates. In the first regression model, these variables are regressed on overall motivation. As the results show, choice does not have any significant effect on overall motivation (*B* = 1.56, *p* = 0.53). Thus, H1a is supported. As the second regression model indicates, choice does not have any direct effect on performance (*B* = −1.08, *p* = 0.64). Index of indirect effect of choice on performance through overall motivation is insignificant (Effect = 0.82, 95% CI [-0.10, 1.81]). Thus, hypothesis H1b is supported. Table 3 provides the results.

**TABLE 3:** OLS regression bias-corrected analysis of choice effect on overall motivation and performance.

			Conf. interval	
Overall motivation regressed on	*B*	*SE B*	Lower 95%	Upper 95%
Constant	23.21	2.40	18.47	27.95
Choice	1.56	0.89	–0.19	3.32
Non-salient reward	1.68	1.08	–0.46	3.81
Salient reward***	4.02	1.10	1.86	6.18
Confidence*	1.36	0.49	0.39	2.32
Competence	0.04	0.51	–0.97	1.05
Interest	0.83	0.35	0.14	1.52
PureGuess*	0.01	0.02	–0.04	0.05
***R*^2^ = 20.79 (*p* < 0.001)**				

			**Conf. interval**	
**Performance regressed on**	***B***	***SE B***	**Lower** **95%**	**Upper** **95%**

Constant	22.40	4.63	13.26	31.54
Overall motivation***	0.52	0.12	0.29	0.75
Choice	–1.08	1.40	–3.85	1.70
Non-salient reward***	6.83	1.71	3.45	10.20
Salient reward***	7.63	1.78	4.11	11.14
Confidence***	–2.73	0.78	–4.28	–1.19
Competence	1.17	0.80	–0.41	2.75
Interest	0.48	0.55	–0.61	1.58
PureGuess	–0.04	0.03	–0.10	0.03
***R*^2^ = 27.58 (*p* < 0.001)**				

*p < 0.05, ***p < 0.001.

To test hypotheses H2a and H2b, we ran a mediation model with salient reward and no reward conditions added. As the results suggest, salient reward improves overall motivation compared to non-salient reward (*B* = 2.34, *p* < 0.05). Thus, H2a is supported. The mediation test shows that the indirect effect of salient reward on performance through overall motivation is significant (Effect = 1.23, 95% CI [0.06, 2.73]). Therefore, H2b is supported. Table 4 shows the results.

**TABLE 4:** OLS regression bias-corrected analysis of effect of salient reward compared to non-salient reward on overall motivation and performance.

			Conf. interval	
Overall motivation regressed on:	*B*	*SE B*	Lower 95%	Upper 95%
Constant	24.89	2.34	20.27	29.52
Salient reward*	2.34	1.09	0.19	4.50
No reward	–1.68	1.08	–3.81	0.46
Choice	1.56	0.88	–0.19	3.32
Confidence**	1.36	0.49	0.39	2.32
Competence	0.04	0.51	–0.97	1.05
Interest*	0.83	0.35	0.14	1.52
PureGuess	0.01	0.02	–0.04	0.05
***R*^2^ = 20.79 (*p* < 0.001)**				

			**Conf. Interval**	
**Performance regressed on**	***B***	***SE B***	**Lower** **95%**	**Upper** **95%**

Constant	29.23	4.68	20.00	38.45
Overall Motivation***	0.52	0.12	0.29	0.75
Salient Reward	0.80	1.73	–2.62	4.22
No Reward***	–6.82	1.71	–10.20	–3.45
Choice	–1.08	1.40	–3.85	1.70
Confidence	–2.73	0.78	–4.28	1.19
Competence*	1.17	0.80	–0.41	2.75
Interest	0.48	0.55	–0.61	1.58
PureGuess	–0.04	0.03	–0.10	0.03
***R*^2^ = 27.58 (*p* < 0.001)**				

*p < 0.05, **p < 0.01, ***p < 0.001.

To test hypotheses H3a and H3b, we ran a mediation model with both salient and non-salient rewards added and no reward condition was considered as the reference group. This model is the same as the model presented in Table 3. Therefore, we do not present it again. The only difference is that salient reward is added as the independent variable and other variables are added as covariates. As the results suggest, salient reward improves overall motivation compared to no-reward condition (*B* = 4.02, *p* < 0.001). Thus, H3a is supported. The mediation test shows that the indirect effect of salient reward on performance through overall motivation is significant (Effect = 2.11, 95% CI [0.85, 3.70]). Therefore, H3b is supported.

## Discussion

Reward and choice are two widely used motivational mechanisms that have proved to positively affect motivation and performance in many different contexts (Hackman and Oldham, 1976; Atkinson et al., 2001; Gagné and Bhave, 2011; Garbers and Konradt, 2014). From the perspective of behavioral psychology and standard theories in economics, these two motivational mechanisms are additive and can reinforce each other (Hamner and Foster, 1975). In contrast, cognitive evaluation, self-determination, and motivational crowding-out theories argue that the controlling nature of some types of external rewards especially performance-contingent ones can undermine the positive effect of choice and decrease motivation and performance (Deci and Ryan, 1985; Frey and Jegen, 2001; Gagné, 2014).

In line with attributional theories and focusing on the notion of perceived locus of causality, motivational congruence effect attempts to reconcile these two contrasting views by providing a holistic approach which combines the effect of different motivational mechanisms, including internal and external ones and their match with each other and the environment in which they are administered.

In order to examine the propositions of motivational congruence theory, the current study tests the effect of reward salience and choice on overall motivation and performance in a controlling context. These effects have not been examined in previous studies. The results show that in a controlling context, choice does not have any significant effect, but salient reward does improve overall motivation and performance because of its match with the environment. It is important to note that controlling elements such as the need for attention and performance and time pressure are an integral part of many tasks. Consistent with the motivational congruence theory, the results of the current study highlight the importance of creating a fit between the motivational mechanisms and the context. Accordingly, if the environment is controlling, choice is unlikely to create the anticipated benefits while salient reward positively influences motivation and performance.

The results of this study and similar studies in the reward literature highlight the importance of taking a new look at the negative effects of extrinsic rewards as predicted in the undermining literature. That is, if administered properly, even salient external rewards can improve overall motivation and performance (Goswami and Urminsky, 2017; Woolley and Fishbach, 2018; Hendijani and Steel, 2020; Zhang et al., 2020). In fact, under certain conditions as can be seen in this study and previous similar ones (Madan and Spetch, 2012), the salience of the reward rather than its absolute value contributes to performance improvement. The current study’s results also suggest that choice, a factor that can contribute to autonomy as an underlying element of intrinsic motivation may not always improve motivation and performance, especially if it is not congruent with the context in which it is administered.

From a practical standpoint, the results of this study indicate that creating congruence between the motivational mechanisms and the context is highly important. Motivational mechanisms such as extrinsic monetary reward and choice are pervasive in different practical settings. They are widely recommended and used in education, work, and health-related areas. These results show that the effectiveness of these motivational mechanisms depends on their congruence with the context. Offering choice is generally recommended as a beneficial and effective motivational mechanism. However, as the results of this study indicate if it is given in a controlling context, it can lose its effectiveness and have no or negative effects on motivation and performance. On the other hand, it is generally recommended to use non-salient external reward compared to salient ones. Salient rewards are mostly regarded as detrimental to motivation. The results of this study indicate that under conditions of control, salient rewards are more appropriate for motivation and performance improvement and can have more beneficial effects compared to non-salient and no-reward conditions.

In total, the findings emphasize the importance of the congruence between different motivational mechanisms and the context in creating total motivation and directing behavior (Kruglanski et al., 2002). As commonly used, different motivational mechanisms occur or are applied in combination with one another (Covington and Müeller, 2001; Breugst et al., 2020). Thus, it is important to mix them appropriately so that they can create a consistent effect on individual’s perceived locus of causality, leading to overall motivation and performance improvement.

## Future Research and Limitations

There are several areas of future research. Considering that perceived locus of causality appears to underpin the combined effect of different motivational mechanisms, examining the separate and joint effect of different motivational mechanisms on the perceived locus of causality can shed light on the underlying mechanisms of motivation and performance improvement. Regarding this study, measuring perceived locus of causality would allow us to confirm whether the joint effect of reward salience and choice on motivation and performance is through this mechanism. In addition, examining the study’s hypotheses in work settings would increase the generalizability of the study results, though this is naturally a later stage of investigation as invariably it requires a quasi-experimental design (Kozlowski, 2012).

In addition, individual differences should prove important to understanding motivational interactions. The motivational congruence effect is dependent on competition among internal and external loci of causality. Those with strong locus of control related traits may override environmental manipulations (Ryan and Connell, 1989). In particular, tests such as the Academic Motivation Scale (Vallerand et al., 1992), which helps assess the degree people are intrinsically or extrinsically motivated, should moderate the observed effect. Finally, this study examined the effect of motivational congruence effect in a controlling context. It is suggested that this effect be tested in future studies using an experimental design with both controlling and non-controlling conditions. This can help in expanding the results and testing the effect of control in reference to a base-line (i.e., no-control) condition in one single experimental setting.

## Data Availability Statement

The datasets presented in this study can be found in online repositories. The names of the repository/repositories and accession number(s) can be found below: https://osf.io/dakgh/?view_only=df27077fed31443ab1038e8e0e81d6be.

## Ethics Statement

The studies involving human participants were reviewed and approved by the Conjoint Faculties Research Ethics Board (CFREB) at the University of Calgary. The patients/participants provided their written informed consent to participate in this study.

## Author Contributions

All authors listed have made a substantial, direct, and intellectual contribution to the work, and approved it for publication.

## Conflict of Interest

The authors declare that the research was conducted in the absence of any commercial or financial relationships that could be construed as a potential conflict of interest.

## Publisher’s Note

All claims expressed in this article are solely those of the authors and do not necessarily represent those of their affiliated organizations, or those of the publisher, the editors and the reviewers. Any product that may be evaluated in this article, or claim that may be made by its manufacturer, is not guaranteed or endorsed by the publisher.

## Footnotes

1
www.empirisoft.com
