# Supplementary material for: Reward Salience and Choice in a Controlling Context: A Lab Experiment
Source: Front Psychol. 2022 Apr 25;13:862152. doi: 10.3389/fpsyg.2022.862152 (PMC9083268; doi:10.3389/fpsyg.2022.862152)
Supplement: Supplementary file 1 [file Table_1.pdf]

## Appendix I

Table 1-AI

*Choice Manipulation*

Choice condition

“Select the Test of Your Own Choice! In the other group, I chose the type of memory test that the participants took. They had no choice in selecting the type of test they wanted to take. But, in this group, you are given the choice to select which memory test you want to take. The following part describes all the details of the two types of memory tests. Please read the descriptions carefully and make your selection. After making your selection, you will be directed to the test of your own choice."

No-choice condition

"In the other group, I let people choose the memory test that they wanted to take, but I am not doing that in this group. Instead, I have assigned you the test that you SHOULD take. Click “Continue” to start the test that is assigned to you."

Table 2-AI

*Test Descriptions in the Choice Condition*

“There are two types of memory tests. Both tests have an acceptable level of accuracy and are able to give a validated measure of your memory skills. Please select which one you would prefer to take:

Test A: This test is a well-designed memory test developed by an organization that was established by North American scientists in the 1950's. In 2007, the organization opened its first international location in London. The test can measure explicit memory skills of individuals. It has been commonly used by researchers in different countries including Canada for more than 70 years and its validity is proven.

Test B: This test is a computer-adaptive memory test primarily developed by a group of management scientists located in Reston, Virginia. The test is now widely used in many countries around the world including Australia, Canada, and the United States of America. It can provide a validated measure of different aspects of a person's memory skills. While its validity is proven after more than 60 years of use, it continues to perform further validity studies to verify that the test provides a good measure of the person's memory skills.”

## Appendix II

Items and Reliabilities for the Self-Reported Measure of Overall Motivation

Factor 1: Interest/Enjoyment ($\alpha = 0.72$)

I thought the memory test was very interesting.

I thought this was a boring test. (R)

I enjoyed taking the memory test very much.

Factor 2: Perceived Effort ($\alpha = 0.75$)

I did not try hard to do this activity very well. (R)

I put a lot of effort into this activity.

Factor 3: Value ($\alpha = 0.80$)

I believe this test was of some value to me.

I believe this test was beneficial to me.

Factors and Factor Loadings

N = 195

| | Components | | |
|---|---|---|---|
| | 1 | 2 | 3 |
| I thought the memory test was very interesting. | **.80** | .01 | .31 |
| I thought this was a boring test. (R) | **-.77** | .32 | .02 |
| I enjoyed taking the memory test very much. | **.69** | .01 | .50 |
| I did not try hard to do this activity very well. (R) | -.05 | **-.91** | -.04 |
| I put a lot of effort into this activity. | .16 | **.81** | .31 |
| I believe this test was of some value to me. | .19 | -.12 | **.84** |
| I believe this test was beneficial to me. | .20 | -.21 | **.87** |

## Appendix III

## Results of Mediated Tests without Control Variables

The results of the regression models without the control variables are presented here. As mentioned before, these results are consistent with the results of the tests with control variables. In the first model choice is regressed on overall motivation and performance. As the results of the first regression model indicate, choice does not have any significant effect on overall motivation ($B = 2.57$, $p = 0.19$). Thus, H1a is supported. As the second regression model shows, choice does not have any direct effect on performance ($B = -0.17$, $p = 0.66$). Index of indirect effect of choice on performance through overall motivation is insignificant (Effect = 0.63, 95% CI [-0.30, 1.67]). Thus, hypothesis H1b is supported. Table 1-AIII provide the results of the analysis.

Table 1-AIII

*OLS Regression Bias-Corrected Analysis of Choice Effect on Overall Motivation and Performance*

| | | | Conf. Interval | |
|---|---|---|---|---|
| Overall motivation regressed on: | *B* | *SE B* | Lower 95% | Upper 95% |
| Constant | 43.28 | 1.96 | 39.42 | 47.14 |
| Choice | 2.57 | 1.95 | -1.27 | 6.41 |
| Non-salient Reward** | 6.63 | 2.39 | 1.92 | 11.35 |
| Salient Reward*** | 13.97 | 2.38 | 9.27 | 18.67 |
| $R^2 = 16.27$ ($p < 0.001$). | | | | |
| | | | Conf. Interval | |
| Performance regressed on: | *B* | *SE B* | Lower 95% | Upper 95% |
| Constant | 23.67 | 2.67 | 18.43 | 28.91 |
| Overall Motivation*** | 0.25 | 0.05 | 0.14 | 0.35 |
| Choice | -0.34 | 1.39 | -3.08 | 2.41 |
| Non-salient Reward*** | 5.97 | 1.73 | 2.54 | 9.40 |
| Salient Reward*** | 6.86 | 1.82 | 3.09 | 10.46 |
| $R^2 = 25.63$ ($p < 0.001$). | | | | |

*Note*. * $p < 0.05$, ** $p < 0.01$, *** $p < 0.001$.

To test hypotheses H2a and H2b, we ran a mediation model with salient reward and no reward conditions added. As the results suggest, salient reward improves overall motivation compared to non-salient reward ($B = 7.34$, $p < 0.05$). Thus, H2a is supported. The mediation test shows that the indirect effect of salient reward on performance through overall motivation is significant (Effect = 1.82, 95% CI [0.55, 3.57]). Therefore, H2b is supported. Table 2-AIII shows the results of the analysis.

Table 2-AIII

*OLS Regression Bias-Corrected Analysis of Effect of Salient Reward Compared to Non-Salient Reward on Overall Motivation and Performance*

| Overall motivation regressed on: | *B* | *SE B* | Conf. Interval Lower 95% | Conf. Interval Upper 95% |
|---|---|---|---|---|
| Constant | 49.92 | 1.91 | 46.14 | 53.69 |
| Salient Reward** | 7.34 | 2.38 | 2.64 | 12.04 |
| No Reward** | -6.63 | 2.39 | -11.35 | -1.92 |
| Choice | 2.57 | 1.95 | -1.27 | 6.41 |
| $R^2 = 16.27$ ($p < 0.001$). | | | | |
| **Performance regressed on:** | ***B*** | ***SE B*** | **Conf. Interval Lower 95%** | **Conf. Interval Upper 95%** |
| Constant | 29.64 | 2.95 | 23.84 | 35.44 |
| Overall Motivation*** | 12.33 | 2.49 | 7.41 | 17.25 |
| Salient Reward | 0.76 | 1.74 | -2.67 | 4.19 |
| No Reward*** | -5.97 | 1.74 | -9.40 | -2.54 |
| Choice | -0.34 | 1.39 | -3.08 | 2.41 |
| $R^2 = 25.63$ ($p < 0.001$). | | | | |

*Note*. * $p < 0.05$, ** $p < 0.01$, *** $p < 0.001$.

To test hypotheses H3a and H3b, we ran a mediation model with both salient and non-salient rewards added and compared to the no reward condition as the reference group. This model is the same as the model presented in Table 1-AIII. Therefore, we do not present this model again. As the results suggest, salient reward improves overall motivation compared to the no reward condition ($B = 13.97$, $p < 0.001$). Thus, H3a is supported. The mediation test shows

that the indirect effect of salient reward on performance through overall motivation is significant (Effect = 3.46, 95% CI [1.65, 5.78]). Therefore, H3b is supported.
